# Matrix Metalloproteinases in Hepatocellular Carcinoma: Mechanistic Roles and Emerging Inhibitory Strategies for Therapeutic Intervention

**DOI:** 10.3390/cancers18020288

**Published:** 2026-01-17

**Authors:** Alexandra M. Dimesa, Mathew A. Coban, Alireza Shoari

**Affiliations:** Department of Cancer Biology, Mayo Clinic Comprehensive Cancer Center, 4500 San Pablo Rd., Jacksonville, FL 32224, USA; dimesa.alexandra@mayo.edu (A.M.D.); coban.mathew@mayo.edu (M.A.C.)

**Keywords:** matrix metalloproteinases (MMPs), hepatocellular carcinoma (HCC), extracellular matrix remodeling, MMP inhibitors, targeted cancer therapy

## Abstract

Liver cancer, also known as hepatocellular carcinoma (HCC), remains a major global health concern, with high mortality driven by late-stage diagnosis, limited treatment efficacy, and frequent therapeutic resistance. Matrix metalloproteinases (MMPs), a large family of zinc-dependent endopeptidases, are central to the biological processes that drive liver tumor initiation and progression. By degrading and reorganizing extracellular matrix components, MMPs facilitate tumor expansion, tissue invasion, and metastatic dissemination. In addition, these enzymes regulate the availability of growth factors, cytokines, and chemokines, thereby influencing angiogenesis, inflammation, immune cell recruitment, and the development of an immunosuppressive tumor microenvironment. Aberrant expression or activity of multiple MMP family members is consistently associated with aggressive clinicopathologic features, including vascular invasion, increased metastatic potential, and reduced patient survival, highlighting their promise as prognostic markers and actionable therapeutic targets. Past attempts to modulate MMP activity were hindered by broad inhibition profiles and dose-limiting toxicities, underscoring the need for improved specificity and delivery strategies. Recent advances in molecular design, biologics engineering, and nanotechnology have revitalized interest in MMP targeting by enabling more selective, context-dependent modulation of proteolytic activity. Preclinical studies demonstrate that carefully tuned MMP inhibition can limit tumor invasion, enhance anti-angiogenic responses, and potentially improve the efficacy of existing systemic therapies, including immuno-oncology agents. This review synthesizes current knowledge on the multifaceted roles of MMPs in HCC pathobiology and evaluates emerging therapeutic strategies that may finally unlock the clinical potential of targeting these proteases.

## Simple Summary

Liver cancer, particularly hepatocellular carcinoma, is a highly aggressive disease with limited treatment options and poor outcomes for many patients. A major reason for cancer spread and treatment resistance is the ability of tumor cells to reshape their surrounding tissue environment. Matrix metalloproteinases (MMPs) are enzymes that play a central role in this process by remodeling tissue structure, promoting tumor invasion, altering blood vessel formation, and influencing immune responses. This review summarizes current knowledge on how these enzymes contribute to liver cancer development and progression and discusses why earlier drugs targeting them were unsuccessful. Importantly, it highlights recent advances that allow more precise and safer targeting of specific enzymes or pathways. These emerging strategies may help improve existing therapies, reduce tumor recurrence, and guide future research toward more effective and personalized treatment approaches for liver cancer.

## 1. Introduction

Liver cancer represents a major global health challenge, ranking as the sixth most diagnosed cancer and the third leading cause of cancer-related mortality worldwide [1]. Hepatocellular carcinoma (HCC) accounts for approximately 80–90% of primary liver cancers [2]. Together, these malignancies contribute to more than 900,000 new cases and 800,000 deaths annually [3]. The incidence is especially high in East and Southeast Asia, sub-Saharan Africa, and certain regions of Europe, primarily due to chronic hepatitis B virus (HBV) and hepatitis C virus (HCV) infections [4]. In contrast, Western countries are witnessing increasing cases associated with metabolic syndrome, non-alcoholic fatty liver disease (NAFLD), and alcohol-related liver cirrhosis [5]. Despite advancements in surveillance programs and multimodal treatment strategies, the prognosis for patients with advanced disease remains dismal, with five-year survival rates rarely exceeding 15–20% [6]. Current therapeutic options include curative approaches such as surgical resection and liver transplantation, as well as loco-regional therapies (radiofrequency ablation, transarterial chemoembolization) and systemic therapies (tyrosine kinase inhibitors like sorafenib and lenvatinib, and immune checkpoint inhibitors such as atezolizumab combined with bevacizumab) [7]. However, recurrence, drug resistance, and limited patient eligibility significantly reduce their overall impact, and this therapeutic gap emphasizes the need to explore novel molecular targets that directly influence tumor initiation, progression, and metastatic spread [8].

**Figure 1:**
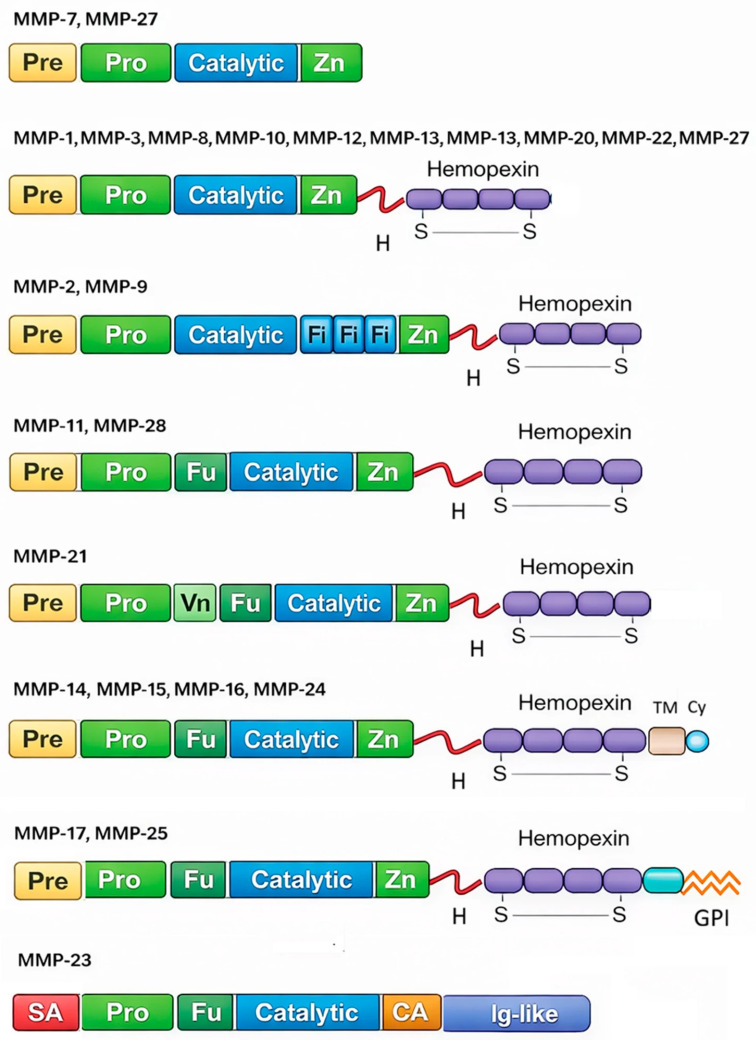
Domain organization and structural diversity of the human matrix metalloproteinase (MMP) family. Schematic representations illustrate the modular architecture of representative MMP subgroups, highlighting conserved and variable domains. Most MMPs contain an N-terminal signal peptide (Pre), a pro-domain (Pro) with the cysteine switch motif, a catalytic domain coordinating the active-site Zn^2+^ ion (Zn), and, except for matrilysins, a C-terminal hemopexin domain composed of four β-propeller blades linked via a flexible hinge. Gelatinases (MMP-2 and MMP-9) uniquely harbor three fibronectin type II (Fi) repeats within the catalytic domain. Furin-activatable MMPs (e.g., MMP-11, -14, -15, -16, -21, -24, -25, -28) contain a furin recognition sequence (Fu) enabling intracellular activation. Membrane-type MMPs are anchored either by a transmembrane (TM) domain with a cytoplasmic tail (Cy) or via a GPI anchor. MMP-23 is structurally distinct, lacking the hemopexin domain and instead containing a signal anchor (SA), cysteine array (CA), and an Ig-like domain. This diversity underlies differences in substrate specificity, localization, and biological function across the MMP family.

Matrix metalloproteinases (MMPs) are a family of zinc-dependent endopeptidases comprising over 20 members, broadly classified into collagenases, gelatinases, stromelysins, matrilysins, and membrane-type MMPs (Figure 1) [9]. Initially recognized for their role in degrading extracellular matrix (ECM) proteins, MMPs are now known to regulate diverse biological processes, including activation of latent growth factors, modulation of cytokine and chemokine activity, angiogenesis, epithelial–mesenchymal transition (EMT), and immune surveillance [10]. Under physiological conditions, MMP activity is tightly controlled by tissue inhibitors of metalloproteinases (TIMPs), but in pathological states such as cancer, dysregulated MMP expression and activity creates a permissive microenvironment that fosters tumor growth, invasion, and metastasis [11]. In the context of HCC, several MMPs, most notably MMP-2 and MMP-9 (gelatinases), MMP-7 (matrilysin), and MMP-14 (MT1-MMP), have been consistently linked to aggressive disease behavior [12]. Elevated expression of these proteases correlates with vascular invasion, intrahepatic and extrahepatic metastasis, and poor overall survival [13]. Mechanistically, MMPs facilitate basement membrane degradation, promote angiogenesis via vascular endothelial growth factor (VEGF) pathway modulation, and regulate EMT by altering cell–cell and cell–matrix interactions [13]. Furthermore, MMPs influence the immunosuppressive tumor microenvironment by processing immune-modulatory molecules, thereby contributing to resistance against systemic therapies [14].

Given their multifaceted roles in tumor progression, MMPs have long been regarded as attractive therapeutic targets. However, early attempts to block their activity using broad-spectrum inhibitors (e.g., batimastat, marimastat) were largely unsuccessful due to poor selectivity, dose-limiting musculoskeletal side effects, and lack of clear patient stratification [15]. The recognition that not all MMPs are tumor-promoting—and that some may even exert protective or anti-tumor functions—has reshaped the field toward more selective strategies [16]. Current efforts focus on designing next-generation inhibitors with enhanced specificity, employing protein engineering approaches such as engineered TIMPs and monoclonal antibodies [17,18,19], as well as exploring novel modalities including nanobodies [20,21], RNA interference [22], and nanoparticle-mediated delivery systems [23].

While MMPs have long been investigated as therapeutic targets in cancer, early clinical failures revealed the limitations of non-selective inhibition strategies. These experiences have reshaped the field toward a new paradigm in which MMPs are viewed as context-dependent effectors whose pathological functions emerge only within specific inflammatory, stromal, and therapeutic settings. Accordingly, this review is structured to highlight not only the diverse biological roles of MMPs in HCC, but also how improved mechanistic insight is enabling selective, temporally controlled, and microenvironment-aware targeting strategies with renewed translational potential.

Thus, a comprehensive evaluation of the biological roles of MMPs in HCC and an updated review of emerging inhibitory strategies are essential for identifying new therapeutic opportunities. This review aims to (i) summarize the mechanistic contributions of MMPs to HCC pathogenesis, (ii) critically analyze past and ongoing attempts at pharmacological inhibition, and (iii) highlight innovative approaches that may overcome previous barriers and pave the way toward clinically effective MMP-targeted therapies.

## 2. Matrix Metalloproteinases in HCC Biology

### 2.1. Tumor Initiation and Chronic Inflammation

**Figure 2:**
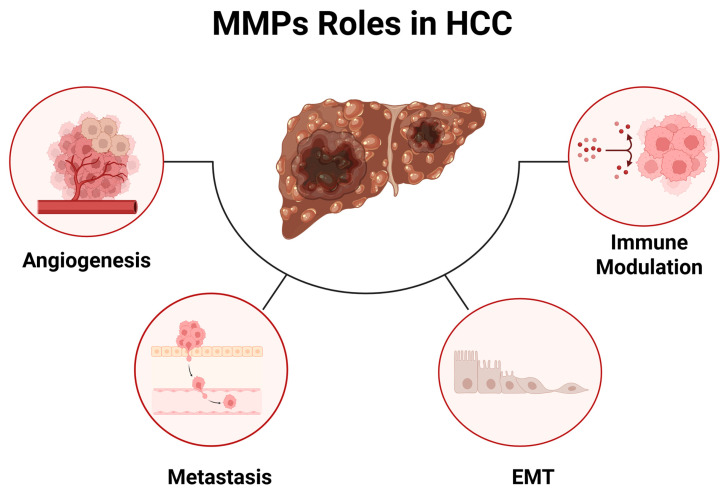
Multifaceted roles of MMPs in hepatocellular carcinoma (HCC) progression. Schematic overview illustrating how dysregulated MMP activity contributes to key hallmarks of HCC. MMPs promote tumor angiogenesis by remodeling the extracellular matrix and releasing pro-angiogenic factors, facilitate local invasion and distant metastasis through basement membrane degradation and stromal remodeling, and drive epithelial–mesenchymal transition (EMT) by disrupting cell–cell junctions and activating EMT-associated signaling pathways. In parallel, MMPs modulate the tumor immune microenvironment by processing cytokines, chemokines, and immune checkpoint-related molecules, thereby influencing immune cell recruitment and function. Collectively, these interconnected processes position MMPs as central regulators of HCC progression and therapeutic response.

MMPs play a central role in the earliest phases of hepatocarcinogenesis by remodeling the chronically injured liver microenvironment (Figure 2). During persistent HBV or HCV infection, alcoholic liver disease, aflatoxin exposure, or metabolic dysfunction-associated steatohepatitis, hepatocytes undergo cycles of necrosis and regeneration, accompanied by activation of Kupffer cells, infiltrating monocytes and neutrophils, hepatic stellate cells, and fibroblasts [24,25,26]. These cells secrete pro-fibrotic cytokines, growth factors, and a broad spectrum of MMPs whose activities interact to shape a matrix-rich, inflamed, and growth-factor-laden microenvironment conducive to malignant transformation [27]. The collagenases MMP-1, MMP-8, and MMP-13 initiate degradation of fibrillar collagens that accumulate during fibrosis, changing tissue stiffness and promoting release of mitogens such as TGF-β1. PDGF and HGF [28]. MMP-1 produced by stromal fibroblasts and macrophages supports expansion of dysplastic nodules [29], while MMP-13 contributes to the remodeling of fibrotic septa that surround emerging lesion clusters [30]. MMP-8, although traditionally a neutrophil enzyme, interacts reciprocally with TGF-β1 in the injured liver, enhancing fibrogenesis and early EMT programming [31]. Parallel to this, the gelatinases MMP-2 and MMP-9 progressively increase from chronic hepatitis to cirrhosis and finally to cancer. By degrading basement membrane components such as collagen IV and laminin, they facilitate pre-neoplastic hepatocyte proliferation and liberate matrix-bound growth factors that reinforce pro-tumorigenic signaling [32]. As fibrosis advances, the imbalance between MMPs and TIMPs creates a dynamic yet pathological ECM landscape that supports clonal evolution and the emergence of malignant phenotypes [33].

### 2.2. Angiogenesis and Vascular Remodeling

Angiogenesis is a defining feature of HCC, and MMPs are among its principal molecular drivers. The dense, hypoxic cirrhotic liver induces HIF-1α-mediated transcription of MMP-2 and MMP-9, which initiate the angiogenic switch by mobilizing VEGF-A, bFGF, and other pro-angiogenic factors from the ECM [34,35]. MMP-9 is strongly associated with neovascular sprouting, sinusoidal capillarization, and microvascular invasion, making it one of the most reliable markers of aggressive tumor biology [36]. By degrading basement membranes, gelatinases facilitate endothelial migration, prune vascular channels, and pave paths for tumor cell intravasation [37]. The collagenases MMP-1 and MMP-13 further loosen the interstitial matrix and promote angiogenic remodeling around tumor nodules. The membrane-type MMPs, especially MMP-14, play a complementary role by coordinating pericellular proteolysis at the interface between hepatoma cells and endothelial structures [38,39]. In addition to generating pro-angiogenic signals, several MMPs produce fragments with anti-angiogenic activity, such as angiostatin or endostatin; however, in the tumor context these inhibitory pathways are insufficient to overcome the dominant pro-angiogenic forces. Through their combined structural and biochemical effects, MMPs sculpt the abnormal, tortuous vascular networks characteristic of HCC and contribute to both intrahepatic spread and early extrahepatic dissemination.

### 2.3. Invasion, Migration, and EMT

The transition of hepatocytes from epithelial to mesenchymal, motile phenotypes is critically regulated by an integrated network of MMPs [40]. EMT in HCC is driven by TGF-β, inflammatory mediators, hypoxia, and mechanical signals within the cirrhotic matrix, but MMPs serve as the primary proteolytic executors of this transformation [10]. MMP-7 promotes EMT by cleaving E-cadherin and releasing β-catenin, thereby activating Wnt signaling and dismantling epithelial cell junctions [41]. Stromelysins such as MMP-3, MMP-10, and MMP-11 modify the ECM, reorganize integrin engagement, and support activation of EMT transcription factors including Snail, Slug, Twist, and ZEB family members [13,42,43]. The membrane-bound MMP-14 is the principal mediator of pericellular collagen degradation and is indispensable for directional migration through the dense fibrotic architecture of cirrhotic liver. By activating pro-MMP-2, reorganizing collagen fibrils, and localizing to invadopodia, MMP-14 endows hepatoma cells with the mechanical capacity to penetrate matrix barriers [44,45]. Other MT-MMPs, including MMP-15, MMP-16, and MMP-24, extend this proteolytic cascade, further promoting three-dimensional invasion [46,47,48]. Meanwhile, MMP-13 and MMP-1 reshape fibrillar collagen networks, reducing physical confinement and enhancing both collective and single-cell invasive modes [49,50]. Through these mechanisms, the MMP family orchestrates the breakdown of structural constraints, the reprogramming of adhesion systems, and the activation of EMT circuitries that collectively drive HCC invasion and metastasis.

### 2.4. Immune Modulation and Tumor–Immune Microenvironment

MMPs exert profound effects on immune composition, immune signaling, and immune evasion in hepatocellular carcinoma and via proteolytically processing cytokines, chemokines, and their receptors, MMPs alter the chemotactic signals that guide the recruitment of lymphocytes, macrophages, and neutrophils [51]. MMP-9 is a pivotal immune-modulating enzyme; it activates latent TGF-β1, which fosters a suppressive microenvironment enriched in regulatory T cells, myeloid-derived suppressor cells, and tumor-associated macrophages [52]. MMP-7 has been shown to proteolytically cleave membrane-bound Fas ligand (FasL), generating soluble FasL and thereby attenuating Fas-mediated apoptotic signaling, which can contribute to tumor cell survival and resistance to immune-mediated apoptosis in cancer cells [53]. Several MMPs, including MMP-14 and MMP-12, reshape chemokine gradients such as those involving CXCL9, CXCL10, and CXCL12, thereby influencing whether CD8^+^ T cells or suppressive myeloid populations dominate the tumor infiltrate [54,55]. Elevated expression of MMP-1, MMP-2, and MMP-9 correlate with exhausted T-cell signatures, sparse effector T-cell infiltration, and attenuated responses to immunotherapies [56,57,58,59]. The dense, crosslinked ECM—formed through chronic fibrosis and continuously remodeled by MMPs and TIMPs—physically impedes T-cell penetration and restricts immune surveillance [60]. Collectively, the immunologic effects of MMPs contribute significantly to immune escape, resistance to checkpoint blockades, and the establishment of an immunosuppressive stromal niche in HCC.

### 2.5. Clinical Correlations and Prognostic Implications

The aggregate expression patterns of MMPs in HCC carry powerful prognostic value [61]. High levels of MMP-1, MMP-2, MMP-7, MMP-9, MMP-13, and MMP-14 in tumor or peritumoral tissues consistently associate with larger tumor size, vascular invasion, poor differentiation, and early recurrence after resection or ablation [26]. Among them, MMP-9 is one of the strongest predictors of recurrence and reduced overall survival, reflecting its fundamental role in angiogenesis, invasion, and immune modulation [62]. MMP-14 expressions align with portal venous infiltration and satellite nodule formation, marking it as a key determinant of invasiveness [63]. Elevated expression of matrix MMP-1, MMP-2 and MMP-7 also associated with aggressive tumor behavior and reduced overall survival, making it a poor prognostic biomarker in patients with hepatocellular carcinoma [29,64,65]. The combined stromal co-expression of multiple MMPs together with TIMP-1 or TIMP-2 defines particularly aggressive molecular subtypes of HCC, often corresponding to EMT-high or progenitor-like transcriptional signatures [13,66,67]. Gene-expression studies reveal that MMP-enriched profiles are tightly linked to vascular-invasive and poorly differentiated tumors, further cementing the clinical relevance of MMP-driven biology. Although early broad-spectrum MMP inhibitors failed clinically due to toxicity and lack of specificity, contemporary insights into selective MMP targeting suggest promising therapeutic potential, particularly in disrupting the MMP–TGF-β axis, inhibiting MT-MMP-mediated matrix tunneling, and alleviating MMP-dependent immune exclusion. Through an integrated network of structural, biochemical, and immunologic functions, the full spectrum of MMP family members exerts a pervasive influence on HCC behavior, making them indispensable elements in understanding and potentially controlling the progression of this malignancy.

Together, the biological mechanisms outlined above establish MMPs as central integrators of tumor invasion, angiogenesis, immune modulation, and therapeutic resistance in HCC. Importantly, these functions are highly context-dependent, shaped by inflammatory cues, stromal interactions, and treatment-induced tissue remodeling. This mechanistic complexity has profound implications for therapeutic targeting, as it explains both the historical failures of broad-spectrum inhibition and the emerging need for selective, temporally controlled, and microenvironment-aware strategies. Building on this biological framework, the following section examines how these insights have informed the development of contemporary MMP-targeted approaches and the rationale behind next-generation therapeutic interventions.

## 3. Targeting Matrix Metalloproteinases in HCCr: From Biology to Therapy

MMPs sit at a pivotal interface between HCC cell-intrinsic programs and the surrounding liver microenvironment, translating inflammatory cues, growth-factor signaling, and tissue-injury responses into extracellular matrix remodeling, angiogenesis, epithelial–mesenchymal transition, and ultimately invasion and metastasis [68]. Among these proteases, the gelatinases MMP-2 and especially MMP-9 repeatedly emerge as context-dependent effectors in HCC models, with their activity shaped not only by tumor expression but also by stromal induction, activation machinery (e.g., MMP-14/TIMP-2-dependent pro-MMP-2 activation), and endogenous inhibitors (TIMPs) [69,70,71]. This biology has motivated multiple therapeutic strategies ranging from direct catalytic blockade to upstream transcriptional suppression and reinforcement of protease–inhibitor balance, including repurposed drugs, natural products, and adjuvant approaches that reprogram post-ablation remodeling and antitumor immunity. At the same time, historical failures of non-selective MMP inhibitors underscore that successful translation requires precision—matching inhibitor selectivity, dosing, and disease context to the protease networks that are truly driving progression in HCC.

**Figure 3:**
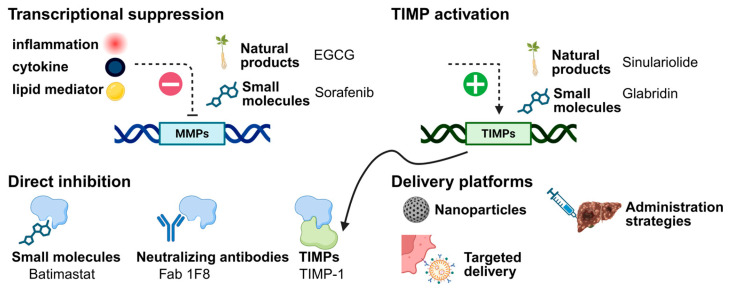
Conceptual overview of therapeutic strategies for modulating MMP activity in HCC. Schematic illustration summarizing the major approaches discussed in Section 3. MMP activity can be reduced through transcriptional suppression driven by inhibition of inflammatory, cytokine, or lipid-mediated signaling pathways, including modulation by natural products and small-molecule inhibitors. Direct inhibition strategies include small-molecule inhibitors and neutralizing antibodies that block MMP catalytic or functional activity. Endogenous regulation can be enhanced through activation or upregulation of TIMPs. Finally, advanced delivery and administration strategies, including nanoparticle-based and targeted delivery platforms, enable spatially and temporally controlled modulation of MMP activity.

Early clinical efforts to therapeutically target MMPs relied heavily on broad-spectrum, hydroxamate-based inhibitors designed to globally block MMP catalytic activity; however, these agents ultimately failed in clinical trials due to limited efficacy and unacceptable toxicity, including musculoskeletal pain and inflammation, as well as unanticipated pro-tumorigenic effects arising from non-selective inhibition of protective or homeostatic MMP functions [72]. These failures revealed that MMPs are not uniformly pro-tumorigenic and that indiscriminate inhibition can disrupt tissue-specific feedback mechanisms, provoke compensatory protease upregulation, and remodel organs such as the liver into metastasis-permissive niches. As a result, the field has shifted away from pan-MMP inhibition toward the development of more refined strategies, including selective inhibitors targeting specific MMPs or activation steps, agents that modulate upstream signaling pathways controlling MMP expression, and approaches that enhance endogenous regulation through TIMPs or microenvironmental reprogramming (Table 1). These complementary therapeutic strategies and their mechanistic points of intervention are summarized schematically in Figure 3.

Collectively, the agents summarized in Table 1 reveal several important patterns that inform current and future strategies for targeting MMPs in HCC. First, most preclinical compounds do not function as direct catalytic inhibitors of MMPs but instead suppress MMP expressions or activity indirectly through modulation of inflammatory signaling, growth factor pathways, or endogenous inhibitors such as TIMPs. Second, most studies converge on the gelatinases MMP-2 and MMP-9 as dominant effectors of invasion, angiogenesis, and metastatic behavior, underscoring their context-dependent importance in HCC progression. At the same time, the breadth of upstream mechanisms highlights a key limitation of many approaches, namely their lack of isoform specificity and reliance on broader pathway inhibition, which may complicate clinical translation. Together, these data support the concept that effective MMP targeting in HCC is more likely to arise from selective, temporally controlled, and microenvironment-aware strategies rather than from broad enzymatic blockade.

**Table 1:** Preclinical pharmacologic agents target MMPs in HCC cell models.

Compound	Target MMP(s)	Primary Mechanism of Action	Cell Line(s) Used	Key Findings	Refs.
Doxycycline	MMP-9, MMP-2	Direct inhibition of gelatinase activity and suppression of HSPG degradation, reduced FGF-2 signaling	HepG2	Reduced vascular invasion, fibrosis, AFP levels, and improved survival	[73,74]
Sorafenib	MMP-2, MMP-9, MMP-3, MMP-7	Suppression of HGF/c-MET, MEK/ERK signaling leading to reduced gelatinase expression	HepG2	Inhibited EMT, migration, and invasion via functional gelatinase blockade	[75,76,77]
Lenvatinib	MMP-1, MMP-2, MMP-7, MMP-9	Transcriptional repression of multiple MMPs and upregulation of TIMP-1/3/4	Hep3B, SMMC-7721	Broad suppression of invasive MMP programs	[35,76]
Pravastatin	MMP-2, MMP-9	Inhibition of MMP-14/TIMP-2 axis preventing pro-MMP-2 activation	Not applicable	Reduced tumor burden and lung metastasis	[78]
Batimastat (BB-94)	MMP-2, MMP-9	Direct catalytic inhibition of gelatinases	H22 (murine hepatoma)	Context-dependent effects, reduced invasion post-ablation but organ-specific pro-metastatic responses	[79]
EGCG	MMP-2, MMP-9	Suppression of gelatinase transcription and secretion under inflammatory stimulation	SK-Hep-1	Near-complete inhibition of MMP-2/-9 even under PMA stimulation	[73]
Curcumin	MMP-9	Selective inhibition of MMP-9 secretion and activity	SK-Hep-1; Huh-7 (comparison)	Strong inhibition of invasion without cytotoxicity	[80]
Resveratrol	MMP-9	NF-κB-dependent transcriptional repression of MMP-9	HepG2	Reduced TNF-α-induced invasion	[81,82]
Pterostilbene	MMP-9	PKC/MAPK/NF-κB and AP-1 inhibition	HepG2	Suppressed EMT, invasion, and experimental metastasis	[81]
Glabridin	MMP-9	NF-κB and AP-1 inhibition with TIMP-1 upregulation	Huh-7, SK-Hep-1	Reduced invasion and tumor growth	[83]
Genipin	MMP-2	p38 MAPK-dependent upregulation of TIMP-1	HepG2, MHCC97L	Selective inhibition of MMP-2-driven invasion	[84]
Oxymatrine	MMP-2, MMP-9	Suppression of p38 MAPK signaling	MHCC97H, HepG2, SMMC-7721	Reduced tumor growth and invasiveness	[85]
Sinulariolide	MMP-2, MMP-9	Inhibition of MAPK, PI3K/Akt, and FAK signaling	HA22T	Coordinated suppression of migration and invasion	[86]
Norcantharidin	MMP-9	ERK/NF-κB inhibition with TIMP-1 induction	Huh-7, SK-Hep-1	Suppressed migration and proteolysis	[87]
Basigin-3	MMP-2, MMP-9	Antagonism of basigin-2-mediated MMP induction	FHCC-98, SMMC-7721	Suppressed invasion and metastasis	[88]

### 3.1. Inflammatory Signaling as a Dominant Upstream Controller of Gelatinase Output

A consistent theme is that inflammatory mediators and tumor microenvironmental stressors can sharply elevate gelatinase production, especially MMP-9, and thereby amplify invasive capacity. Roomi et al., working in SK-Hep-1 cells, directly demonstrated pro-inflammatory stimuli such as phorbol 12-myristate 13-acetate (PMA), tumor necrosis factor alpha (TNF-α), and interleukin-1 beta (IL-1β) markedly induced MMP-9 secretion while exerting minimal or inhibitory effects on MMP-2, highlighting MMP-9 as an inflammation-sensitive invasion effector [73]. In contrast, several inhibitors significantly reduced gelatinase secretion, with doxycycline attenuating MMP-9 and completely blocking the MMP-9 dimer at higher concentrations and notably, the strongest effects in that study came from natural compounds, especially epigallocatechin gallate (EGCG) and a multi-component nutrient mixture containing vitamin C, lysine, proline, EGCG, and micronutrients, which nearly abolished gelatinase secretion even under PMA stimulation [73].

Several studies reinforced the concept that inflammatory transcriptional programs, particularly nuclear factor kappa-light-chain-enhancer of activated B cells (NF-κB) and Activator Protein 1 (AP-1), sit immediately upstream of MMP-9 induction and are therefore tractable points of intervention. In a model relevant to alcohol-associated carcinogenesis, Yeh et al. showed that acetaldehyde potently drove MMP-9 transcription and invasion in HepG2 cells, and that hesperidin blocked this axis by suppressing NF-κB and AP-1 activation through inhibition of inhibitor of kappa B (IκB) phosphorylation/degradation and p38/(c-Jun N-terminal kinase) JNK signaling, with extracellular signal-regulated kinase (ERK) spared, yielding selective suppression of MMP-9 and invasion [89]. A closely aligned cytokine-driven invasion model was presented by Yu et al., where TNF-α induced MMP-9 and Matrigel invasion in HepG2 cells, while resveratrol reduced MMP-9 at the mRNA, protein, and activity levels via NF-κB inhibition; importantly, NF-κB blockade phenocopied resveratrol, supporting causality [82]. Ordóñez et al. extended this logic to IL-1β-stimulated HepG2 cells, showing melatonin selectively reduced MMP-9 (with no effect on MMP-2 or TIMP-2), lowered the MMP-9/TIMP-1 ratio by upregulating TIMP-1, and inhibited invasion through suppression of IKK/IκBα phosphorylation and p65 nuclear translocation [90]. In parallel, multiple natural products converged on NF-κB/AP-1 repression to suppress MMP-9. *Terminalia catappa* leaf extracts reduced invasion in Huh7 cells with transcriptional repression of MMP-9 while upregulating TIMP-1 through inhibition of NF-κB and AP-1 nuclear translocation and promoter binding [91], while matrine similarly suppressed MMP-9 expression and invasion in SMMC-7721 cells via NF-κB inhibition, again supported by NF-κB inhibitor phenocopy [92]. These data collectively suggest that inflammation-driven MMP-9 programs are a recurrent and actionable dependency for HCC invasion, and that compounds targeting these transcriptional nodes can function as context-dependent “MMP inhibitors” even without direct enzymatic inhibition.

### 3.2. Growth Factor and Lipid Mediator Pathways That Converge on MMP-9-Dependent Invasion

Beyond canonical inflammatory stimuli, growth-factor and lipid mediator signaling can activate invasion programs with MMP-9 as a pivotal downstream effector. Park et al. identified the autotaxin (ATX)-lysophosphatidic acid (LPA)-LPA1 axis as clinically elevated in HCC tissues and mechanistically necessary for LPA-driven invasion across multiple HCC cell lines. In this system, LPA selectively induced MMP-9 (not MMP-2), and genetic, antibody, or functional inhibition of MMP-9 abrogated invasion, supporting MMP-9 as the critical effector. Mechanistically, LPA1-driven MMP-9 induction required coordinated phosphoinositide 3-kinase (PI3K)/Akt and protein kinase C delta (PKCδ)- p38 signaling [93].

Ha and colleagues linked hepatocyte growth factor (HGF)/c-MET-driven EMT to gelatinase activity in HepG2 cells, showing that HGF induced EMT, invasion, and strong upregulation of MMP-2 and MMP-9, all of which were suppressed by sorafenib [75]. Notably, direct gelatinase inhibition using neutralizing antibodies, GM6001, or SB-3CT phenocopied sorafenib by blocking EMT, migration, and invasion and dampening downstream MEK/ERK signaling, identifying MMP-2/9 as critical mediators of growth factor-driven invasiveness [75]. These data argue that in some HCC contexts, gelatinase inhibition is not merely a correlation but a mechanistic requirement for EMT and invasion downstream of growth-factor signaling.

### 3.3. Direct and Indirect Suppression of MMP-2/MMP-9 by Natural Products and Nutraceuticals

A substantial portion of the research supports natural products as indirect suppressors of gelatinase expression and function. Some formulations appear to act through direct functional suppression of secreted gelatinase activity, while many act through upstream signaling networks that set gelatinase transcriptional output. Ha et al. reported that Daesungki-Tang (DST) reduced Hep3B invasion with minimal cytotoxicity and suppressed secreted gelatinase activity by zymography, with IC_50_ values in the ~75–87 µg/mL range for MMP-9 and MMP-2, respectively; Magnoliae cortex was highlighted as a dominant active component [94]. A similarly strong anti-invasive phenotype accompanied dual MMP-2/MMP-9 suppression for several defined compounds, including chelerythrine, which reduced both gelatinases at mRNA and protein levels while driving cytoskeletal reorganization, and mechanistically suppressed phosphorylation of focal adhesion kinase (FAK)-PI3K/Akt/mechanistic target of rapamycin (mTOR) and mitogen-activated protein kinase (MAPK) nodes, supporting a signaling-to-gelatinase pathway [95]. Wu et al. described sinulariolide as a marine diterpenoid that reduced MMP-2/MMP-9 activity and protein levels while upregulating TIMP-1 and suppressing urokinase-type plasminogen activator (uPA), linking reduced invasion to inhibition of MAPK/PI3K/Akt and FAK/GRB2 pathways [86]. Chen and colleagues showed that oxymatrine reduced migration and invasion across multiple HCC lines with downregulation of MMP-2 and MMP-9, mechanistically tied to suppression of p38 phosphorylation and supported by p38 inhibitor synergy; these effects translated into reduced xenograft growth with lower MMP-2/MMP-9 and p-p38 levels [85]. Tian et al. further linked inflammatory signaling control to gelatinase output by demonstrating that the COX-2 inhibitor parecoxib reduced invasion and angiogenesis with decreased ERK phosphorylation, lower VEGF, and downregulation of MMP-2 and MMP-9, with concordant xenograft suppression [96]. These studies collectively emphasize that “MMP inhibition” is frequently achieved by dampening upstream kinase pathways that drive transcriptional and post-transcriptional gelatinase production.

Several nutraceutical and plant-derived compounds were framed explicitly as transcriptional inhibitors of MMP-9, often with supportive functional validation. Curcumin selectively suppressed MMP-9 secretion in highly invasive SK-Hep-1 cells and reduced Matrigel invasion by ~70% at 10 µM without impairing viability, while the less invasive Huh-7 line lacked detectable MMP-9 output, reinforcing MMP-9 as a determinant of invasiveness and a targetable dependency [80]. Hsieh et al. showed that glabridin suppressed migration and invasion in Huh7 and SK-Hep-1 cells with a strong reduction in MMP-9 mRNA, protein, and activity, accompanied by TIMP-1 upregulation and blockade of NF-κB/AP-1 nuclear translocation and promoter engagement, supported by reduced ERK/JNK phosphorylation; importantly, glabridin suppressed xenograft growth without overt toxicity [83]. Likewise, Yeh et al. showed that norcantharidin reduced migration and invasion in Huh7 and SK-Hep1 cells by inhibiting ERK phosphorylation and thereby NF-κB binding to MMP-9 and uPA promoters while upregulating TIMP-1 and PAI-1, supporting coordinated restraint of multiple proteolytic nodes [87]. Esculetin also acted as a dual gelatinase suppressor in Hep3B and HepG2 cells, reducing MMP-2/MMP-9 mRNA and activity while increasing TIMP-1/TIMP-2, with an additional mechanistic layer in barrier reinforcement via increased transepithelial electrical resistance and modulation of claudins [97]. Complementing these, diosmetin reduced motility, adhesion, and invasion in SK-HEP-1 and MHCC97H cells with reduced PKC-δ expression, inhibited MAPK phosphorylation, and downregulated MMP-2 and MMP-9, supporting a PKC-δ/MAPK/gelatinase axis [98]. Total flavonoids of *Scutellaria barbata* and *Chrysanthemum indicum* extract both presented a similar mechanistic signature in MHCC97H cells, suppressing MMP-2/MMP-9 while upregulating TIMP-1/TIMP-2 and thereby restoring protease–inhibitor balance as a mechanistic basis for invasion control [99,100].

Not all nutraceutical regulation followed the canonical expectation that MAPK activation increases gelatinase output; Huang et al. reported that β-mangostin reduced MMP-2 and MMP-9 expression and invasion across multiple HCC lines while depending on sustained ERK and JNK activation, where ERK/JNK inhibition or knockdown restored gelatinase expression and invasion. This counterintuitive but mechanistically supported finding underscores that gelatinase transcription can be context-dependent and that pathway activation state does not universally map to MMP induction without considering duration, feedback, and transcription factor wiring [101].

### 3.4. Repurposed Drugs, Perioperative Agents, and Pathway-Targeted Therapeutics Modulating MMP Programs

Several studies highlight that agents not classically categorized as MMP inhibitors can nevertheless restrain invasion and metastasis through MMP axis modulation. Zhang et al. linked propofol exposure to reduced adhesion and invasion in HepG2 cells through a miRNA mechanism. Propofol upregulated miR-199a, leading to reduced MMP-9 activity and protein, while anti-miR-199a reversed MMP-9 inhibition and the anti-adhesive phenotype, establishing a causal miRNA-MMP-9 axis [102]. This suggests perioperative or anesthetic-associated modulation of invasive programs may be biologically plausible and mechanistically specific, even if clinical relevance requires careful contextualization.

Targeted therapies used in advanced HCC also intersected with the MMP/TIMP axis. He et al. compared sorafenib, regorafenib, and lenvatinib in Hep3B and SMMC-7721 cells and found all suppressed migration and invasion, but with distinct MMP signatures. sorafenib reduced MMP-7/10/16, regorafenib reduced MMP-1, and lenvatinib showed broad suppression across MMP-1/2/7/9/10/16. These effects were accompanied by TIMP induction patterns, with sorafenib and lenvatinib increasing TIMP-1/3/4 and regorafenib increasing TIMP-3, overall shifting the proteolytic balance toward inhibition [76]. A complementary, clinically anchored perspective was provided by Hsieh et al., who identified SERPING1 as a tumor-suppressive modulator associated with stage and survival and inducible by sorafenib. Recombinant SERPING1 phenocopied sorafenib by reducing migration and suppressing active MMP-2 and MMP-9 via an ERK-linked cascade, including in sorafenib-resistant models, supporting SERPING1 both as a biomarker and as an indirect lever on gelatinase-driven motility [77]. Together, these studies support the idea that part of the anti-metastatic benefit of TKIs may derive from coordinated rewiring of ECM protease networks, and that resistance states may be identifiable and targetable through nodes that converge on gelatinase regulation.

### 3.5. In Vivo Evidence: Tumor Control, Metastasis Suppression, and Microenvironmental Reprogramming

While many of the HCC MMP-inhibition studies are in vitro, several studies provide substantive in vivo validation that gelatinase targeting can influence tumor progression and dissemination. Taras et al., using an autochthonous chemically induced rat HCC model, showed that pravastatin treatment after tumor establishment reduced tumor burden, improved survival trends, and most strikingly diminished spontaneous lung metastases. Mechanistically, pravastatin reduced MMP-9 activity and nearly abolished MMP-2 activation in tumor and peri-tumoral tissue by suppressing the MMP-14/TIMP-2 activation axis necessary for pro-MMP-2 conversion; it also reduced TIMP-1 expression, which is frequently associated with poorer prognosis in HCC contexts [78].

Elewa and colleagues provided a combined human-correlative and in vivo pharmacologic example centered on MMP-9, linking elevated MMP-9 in HCC patient sera and tissue to a vascular invasion-associated profile and showing that doxycycline in a thioacetamide-induced rat model improved survival, reduced alpha-fetoprotein or AFP, mitigated fibrosis and tissue destruction, restored hepatic heparan sulfate proteoglycan (HSPG) integrity, and reduced fascin and fibroblast growth factor 2 (FGF-2) expression. Their proposed cascade, MMP-9-driven HSPG degradation enabling FGF-2 release and cytoskeletal/invasive remodeling, places MMP-9 in a mechanistically coherent pathway that is both disease-relevant and drug-modifiable in vivo [74]. Pan et al. likewise provided in vivo anti-metastatic evidence for transcriptional MMP-9 suppression. Pterostilbene inhibited TPA-induced MMP-9 transcription via PKC/MAPK/PI3K-Akt blockade and reduced experimental lung metastases after tail-vein injection, with decreased plasma MMP-9 activity and VEGF levels [81]. In an orthotopic model emphasizing invasion within the liver microenvironment, Wang et al. demonstrated genipin reduced intrahepatic invasion and tumor size, mechanistically acting through p38-driven TIMP-1 upregulation leading to suppression of MMP-2 activity without changing MMP-2 abundance, providing an elegant example of selective post-translational control via endogenous inhibitor induction [84]. Liao et al. introduced a conceptually distinct inhibitory strategy by showing that basigin-3 antagonizes pro-invasive basigin-2 through hetero-oligomerization, blunting stromal MMP induction in co-culture and suppressing MMP-2/MMP-9 levels and metastasis in an orthotopic model, highlighting isoform-based suppression of MMP induction rather than direct inhibition [88].

Therapy-induced local injury can paradoxically promote invasive programs, and MMP inhibition appears capable of counteracting this effect in vivo. Jiang et al. modeled insufficient radiofrequency ablation (RFA) and demonstrated that heat exposure enhanced invasion and growth, while batimastat reversed these effects in vitro and, when combined with RFA in vivo, reduced residual tumor growth, limited distant outgrowth, and prolonged survival. Mechanistically, BB-94 reduced MMP-2/MMP-9 activity after RFA and suppressed angiogenesis (VEGF/CD31), collagen deposition, and peri-ablational TGF-β signaling, indicating that MMP inhibition can mitigate the pro-invasive wound-healing-like response elicited by incomplete ablation [103]. Shewarega et al. extended the relevance of MMP inhibition beyond invasion control into immuno-oncology. In an orthotopic immunocompetent model, incomplete cryoablation increased M2-tumor-associated macrophage (TAM) infiltration and MMP-9 expression, while BB-94 reduced MMP-9-expressing cells and M2-TAM accumulation; importantly, the combination induced robust CD8^+^ T-cell infiltration and interferon-gamma (IFN-γ)-producing CD8^+^ cells, suggesting MMP inhibition can reshape the post-ablation immune landscape to favor productive antitumor immunity [104]. These data indicate that in HCC, MMP inhibition may function as a microenvironmental adjuvant strategy to improve outcomes after locoregional therapy by limiting pro-invasive remodeling and enhancing immune cell trafficking.

### 3.6. Cautionary Biology: When Broad-Spectrum MMP Inhibition Backfires

Despite multiple supportive datasets, the broader MMP inhibitor field has long been shaped by the recognition that non-selective inhibition can generate unanticipated, tissue-specific consequences. Krüger and colleagues provided a particularly striking in vivo model and showed that batimastat treatment modestly reduced lung metastases yet robustly promoted liver metastasis in two independent models, including induction of de novo hepatic colonization. Mechanistically, batimastat caused liver-specific upregulation of MMP-2 and MMP-9 even in tumor-free animals, alongside increased hepatic expression of HGF, basic fibroblast growth factor (bFGF), angiogenin, and caspase-1, indicating a host-driven compensatory response that remodels the liver into a metastasis-permissive niche [79]. This work exhibited that the liver is a uniquely reactive organ with strong injury-response programs and that indiscriminate MMP blockade can provoke feedback and microenvironmental rewiring that undermine therapeutic intent. Importantly, this caution does not negate the positive HCC-focused ablation-adjuvant studies using BB-94 [103,104], but it underscores the need for context-specific application, dosing strategies, biomarker guidance, and mechanistic understanding of host responses to MMP inhibition.

### 3.7. Emerging Modalities: Nanomaterials and Resistance-Associated Invasion Programs

Beyond classical small molecules and natural products, newer approaches are beginning to target MMP-linked invasive programs indirectly through broader stress and resistance pathways. Elmetwalli et al. reported that probiotic-derived silver nanoparticles reduced HepG2 viability and induced apoptosis/autophagy with transcriptional downregulation of MMP-9, B-cell lymphoma-2 (BCL-2), mTOR, and alpha smooth muscle actin (αSMA), implicating an AMP-activated protein kinase (AMPK)/mTOR/MMP-9 axis and suggesting nanomaterial-driven modulation of invasion-linked survival networks [105]. Kabir and colleagues provided a clinically relevant resistance framework, identifying caveolin-1 as a driver of pan-TKI resistance and metastatic behavior, and showing that caveolin-1 targeting (siCAV1 or miR-7-5p) reduced migration and invasion while decreasing MMP-9 expression and gelatinolytic activity, particularly in 3D tumor–stroma contexts, with mechanistic links to Signal transducer and activator of transcription 3 (STAT3), ERK, NF-κB, and receptor tyrosine kinase signaling networks known to regulate MMP-9 output [106].These studies suggest that in advanced disease, suppressing MMP-driven invasion may require addressing resistance-associated signaling states and tumor–stroma interactions that sustain gelatinase programs under therapeutic pressure.

Beyond small molecules and pathway-directed agents, next-generation therapeutic modalities offer fundamentally new ways to modulate MMP activity with improved specificity and spatial control. Monoclonal antibodies and engineered protein inhibitors can achieve high isoform selectivity by engaging non-catalytic exosites or blocking substrate recognition, thereby avoiding the off-target effects associated with zinc-chelating inhibitors. Similarly, nanobodies, single-domain antibody fragments derived from camelid antibodies, combine high affinity and specificity with small size and favorable tissue penetration, making them attractive candidates for targeting membrane-type MMPs or tumor-associated gelatinases within the dense liver tumor microenvironment. Importantly, these biologics can function through allosteric inhibition or interference with MMP activation and localization rather than direct catalytic blockade.

Nanomaterial-based approaches further expand the therapeutic landscape by enabling targeted and context-dependent delivery of MMP-modulating agents. Nanoparticles and MMP-responsive drug carriers can be engineered to release payloads selectively within protease-rich tumor regions, enhancing intratumoral exposure while limiting systemic toxicity and host compensatory responses. Although most of these strategies remain at the preclinical stage, proof-of-concept studies demonstrate their ability to suppress invasion, reprogram the tumor microenvironment, and synergize with existing systemic or locoregional therapies.

A major barrier to clinical translation of MMP-targeted strategies has been the lack of robust frameworks linking preclinical efficacy to patient selection and on-treatment monitoring. Future clinical development will require pharmacodynamic biomarkers that reflect MMP activity rather than expression alone. These may include circulating or tissue-based measurements of active gelatinases, cleavage products of extracellular matrix substrates, MMP-responsive imaging probes, or dynamic changes in stromal and immune markers associated with protease activity. Such biomarkers could enable real-time assessment of target engagement, inform dosing schedules, and identify compensatory responses that undermine efficacy. Equally critical is patient stratification. Preclinical studies summarized in Table 1 indicate that MMP dependency is highly context-specific, often emerging in tumors characterized by active inflammation, epithelial–mesenchymal transition, vascular invasion, or immune exclusion. Stratifying patients based on these biological features—rather than tumor stage alone—may enrich those most likely to benefit from MMP modulation. Transcriptomic signatures, circulating protease activity, and microenvironmental features such as fibrosis density or immune infiltration patterns represent promising stratification tools. Finally, the translation of MMP-targeted approaches is most likely to succeed within rational combination strategies. Given the roles of MMPs in invasion, angiogenesis, and immune suppression, selective MMP inhibition may function best as an adjuvant rather than a stand-alone therapy. Combining MMP modulators with immune checkpoint inhibitors, anti-angiogenic agents, or locoregional therapies may suppress adaptive resistance mechanisms and improve therapeutic durability. Together, these considerations outline a clinically actionable pathway that connects preclinical MMP biology to trial design, emphasizing biomarker-guided patient selection, pharmacodynamic monitoring, and mechanism-based combination regimens.

Taken together, these studies support a coherent model in which MMP-9 is frequently the most inducible and invasion-linked gelatinase in HCC, especially under inflammatory, cytokine, lipid mediator, and therapy-induced stress, while MMP-2 often contributes through activation-dependent mechanisms tied to MMP-14/TIMP-2 and through contexts where dual gelatinase activity supports invasion and angiogenesis. Effective strategies therefore include transcriptional repression of MMP-9 via NF-κB/AP-1 blockade, broad upstream pathway suppression that converges on MMP-2/MMP-9 downregulation, and reinforcement of endogenous inhibition through TIMP induction. In vivo studies further argue that MMP targeting can affect not only invasion and metastasis but also post-ablation remodeling and antitumor immunity, while landmark cautionary evidence emphasizes that broad-spectrum inhibition can provoke host compensatory responses, especially in the liver, necessitating careful contextualization, selectivity, and biomarker-guided deployment. Within modern HCC therapy, the intersection between MMP and TIMP regulation suggests that protease networks may be both effectors of response and contributors to resistance, emerging nodes offering conceptually distinct routes to suppress gelatinase-driven invasion without relying solely on pan-MMP catalytic blockade.

## 4. Future Directions

Future progress in targeting MMPs in HCC will depend on embracing the complexity of protease biology while leveraging emerging technologies that allow precise, context-aware intervention. A critical priority is the development and validation of isoform-specific MMP inhibitors that discriminate between tumor-promoting and homeostatic functions. Structural and functional studies increasingly reveal exploitable differences in exosites, activation mechanisms, and protein–protein interactions among MMP family members. Targeting these non-catalytic features, particularly in gelatinases and membrane-type MMPs, offers a path toward high specificity without the dose-limiting toxicities observed with earlier inhibitors. Equally important is the integration of biomarker-driven patient selection into future preclinical and clinical studies. MMP expression and activity vary widely across etiologies, molecular subtypes, and stages of hepatocellular carcinoma. Stratification based on inflammatory signatures, EMT status, vascular invasion markers, or immune exclusion phenotypes may identify patient subsets most likely to benefit from MMP modulation. Circulating MMP levels, activity-based probes, and imaging modalities capable of reporting protease activity in vivo could serve as pharmacodynamic markers to guide dosing, scheduling, and therapeutic combinations.

The therapeutic potential of MMP targeting is likely to be maximized through rational combination strategies. Strong mechanistic links between MMP activity and immune suppression suggest that selective inhibition may enhance the efficacy of immune checkpoint blockade by improving T-cell infiltration, reducing TGF-β signaling, and remodeling the extracellular matrix barrier. Similarly, combining MMP inhibitors with anti-angiogenic agents could blunt adaptive vascular responses and prolong vascular normalization. In the context of locoregional therapies such as radiofrequency ablation or transarterial chemoembolization, transient and localized MMP inhibition may suppress therapy-induced invasion and recurrence, offering a particularly attractive translational opportunity. Advances in targeted delivery platforms represent another promising direction. Nanoparticle-based systems, MMP-responsive drug carriers, and locoregional administration strategies may enable high intratumoral exposure while minimizing systemic toxicity and host compensatory responses. These approaches are especially relevant in HCC, where organ-specific feedback mechanisms have been shown to undermine systemic MMP inhibition. Incorporating MMP-targeted agents into existing interventional oncology workflows could accelerate clinical translation while reducing risk.

Importantly, the documented pro-metastatic and tissue-specific compensatory effects of broad-spectrum MMP inhibitors, particularly within the liver, provide critical guidance for the design of next-generation therapeutic strategies. Past failures underscore that patient selection and biomarker integration will be essential for successful clinical translation. Future trials should incorporate biomarkers that report not only MMP expression but also protease activity, inflammatory status, stromal remodeling, and EMT or immune-exclusion phenotypes to identify contexts in which MMP-driven programs are dominant and therapeutically actionable.

Equally important are considerations of dosing and scheduling. Continuous systemic inhibition, which characterized early clinical trials, may provoke host injury responses and compensatory protease upregulation. In contrast, transient, localized, or peri-procedural MMP modulations such as short-term administration following locoregional therapies may suppress therapy-induced invasion while minimizing systemic toxicity and metastatic niche formation. These insights argue for trial designs that emphasize temporal control, spatial targeting, and adaptive biomarker monitoring rather than prolonged pan-MMP blockade. Future research must continue to dissect the cell type-specific and microenvironmental roles of MMPs in HCC. Distinguishing tumor-cell-intrinsic functions from those mediated by stromal fibroblasts, immune cells, and endothelial compartments will be essential for designing interventions that selectively disrupt malignant programs. Single-cell transcriptomics, spatial proteomics, and functional protease mapping are poised to provide unprecedented resolution into these dynamics. Such insights will inform not only about drug development but also the timing and sequencing of MMP-directed therapies relative to other treatments.

## 5. Conclusions

MMPs occupy a central and multifaceted position in the pathobiology of hepatocellular carcinoma and related primary liver malignancies. Far from acting solely as extracellular matrix-degrading enzymes, MMPs function as dynamic regulators of tumor–stroma interactions, angiogenesis, immune modulation, and metastatic competence within the chronically inflamed and fibrotic hepatic microenvironment. The evidence synthesized in this review demonstrates that dysregulated expression and activation of multiple MMP family members, most prominently gelatinases MMP-2 and MMP-9 and the membrane-type protease MMP-14, are tightly linked to aggressive tumor phenotypes, vascular invasion, immune exclusion, therapeutic resistance, and poor clinical outcomes. These associations are reinforced across experimental systems, human tumor specimens, and clinical correlative studies, underscoring the biological and translational relevance of MMP-driven programs in HCC progression.

At the same time, the historical failure of broad-spectrum MMP inhibitors has fundamentally reshaped our understanding of protease targeting in oncology. Early clinical trials revealed that indiscriminate catalytic inhibition disrupts essential physiological functions and elicits tissue-specific compensatory responses, particularly within the liver, that can paradoxically enhance metastatic susceptibility. These outcomes do not negate the importance of MMPs as therapeutic targets; rather, they highlight the necessity of precision, context awareness, and mechanistic selectivity. The emerging view is that MMPs act as inducible effectors whose pathological functions are tightly coupled to inflammatory signaling, stromal activation, and microenvironmental cues, making them highly amenable to selective and temporally controlled intervention.

Recent advances in molecular design, protein engineering, and drug delivery have revived interest in MMP targeting by enabling strategies that go beyond pan-enzymatic blockade. Isoform-selective small molecules, monoclonal antibodies, engineered TIMPs, nanobodies, and pathway-directed transcriptional suppressors offer the ability to modulate discrete MMP activities while preserving protective or homeostatic functions. Importantly, preclinical studies demonstrate that selective suppression of MMP-9-driven inflammatory and angiogenic programs or MMP-14-mediated pericellular matrix remodeling can limit invasion, normalize the tumor microenvironment, and enhance responsiveness to existing systemic therapies. These findings position MMP modulation not as a stand-alone cytotoxic strategy, but as a powerful means of reprogramming tumor behavior and therapeutic sensitivity.

Collectively, the body of evidence reviewed here supports a conceptual shift in how MMPs are viewed in HCC. Rather than static biomarkers or failed drug targets, MMPs should be understood as dynamic, context-dependent regulators of tumor progression whose activities can be therapeutically redirected. With improved biological insight, rational patient stratification, and integration into combination regimens, next-generation MMP-targeted approaches hold genuine promise for addressing unmet clinical needs in hepatocellular carcinoma and other liver malignancies.

## Data Availability

This study did not involve the generation or analysis of any datasets.
